# Neuronal Differentiation of GBM-Initiating Cells Combined with Elimination of Undifferentiated Cells Preserves Motor Function

**DOI:** 10.3390/cells15060539

**Published:** 2026-03-18

**Authors:** Zhenzhong Chen, Peilin Zou, Toru Kondo

**Affiliations:** Division of Stem Cell Biology, Institute for Genetic Medicine, Hokkaido University, Sapporo 060-0815, Japan

**Keywords:** glioblastoma (GBM), GBM-initiating cell (GIC), dihydroorotate dehydrogenase (DHODH), Isoxazole 9 (ISX9), functional preservation

## Abstract

**Highlights:**

**What are the main findings?**

- Sequential ISX9 and BRQ administration drove neuronal differentiation in GICs and eliminated undifferentiated GICs in a brain tumor.
- ISX9 treatment promoted structural integration with host neurons and improved motor performance in tumor-bearing mice.

**What are the implications of the main findings?**

- The combination can be used for maintaining neuronal functions in patients with GBM.
- Intrathecal injection can be used for delivering non-BBB penetrating chemicals into brain.

**Abstract:**

Glioblastoma (GBM) is an aggressive human malignancy. Recent advances in GBM research have highlighted innovative therapeutic approaches, including the use of small molecules that eliminate GBM in mouse models. However, there are few reports on the restoration of lost neuronal functions in patients. Considering that GBM contains GBM-initiating cells (GICs) with characteristics of both cancer and neural stem cells, we investigated whether GICs could be redirected toward non-tumorigenic neurons to support the preservation of neural function in the brain with GBM. We demonstrated that the neuronal differentiation inducer Isoxazole 9 (ISX9) effectively induced GICs to differentiate into neurons, accompanied by significant changes in their gene expression profiles. The sequential application of ISX9 and the DHODH inhibitor brequinar (BRQ), which successfully eradicated undifferentiated GICs, not only promoted neuronal differentiation but also inhibited GIC tumorigenesis in the mouse brain, leading to prolonged survival and preservation of motor function in tumor-bearing mice. Furthermore, pathological analysis revealed that this combination not only reduced the size of GIC brain tumors but also facilitated the formation of synapse-like structural contacts between GIC-derived cells and host mouse neurons, suggesting remodeling of the tumor–neural interface within the tumor-developed area. Collectively, these findings suggest that the modulation of tumorigenic GIC differentiation may represent a strategy to preserve neural circuit integrity within the tumor-bearing brain.

## 1. Introduction

Since the discovery of stem cells, significant research in regenerative medicine has focused on developing methods to restore tissues lost due to diseases [1,2,3]. Brain disorders can lead to the loss of high-level functions, including cognitive and motor abilities, depending on the affected area, thereby significantly diminishing patients’ quality of life (QOL) [4]. Increasing evidence suggests that cell transplantation, such as dopaminergic neurons for Parkinson’s disease and mesenchymal stem cells for stroke, can substantially restore functions lost by patients [5,6]. In the context of brain tumors, the resection of extensive brain areas is impractical due to the critical nature of tissue function, leading to recurrence from residual cancer cells, including brain cancer stem cells. Consequently, research has primarily focused on developing methods to eradicate these residual cancer cells, while studies on recovering functions lost by cancer patients have largely been confined to rehabilitation [7,8].

Glioblastoma (GBM) is the most malignant form of primary brain cancer and is associated with a median survival of about 15 months [9,10]. Despite continuous improvements in combined treatment strategies involving surgery, chemotherapy, and radiotherapy, patient prognosis has shown little progress over recent decades. A significant challenge in treating GBM is largely attributed to a subpopulation of tumor cells known as GBM-initiating cells (GICs), or GBM stem cells. These cells are highly invasive, retain strong tumorigenicity, resist conventional therapies, and possess neural stem cell (NSC)-like multipotency [11,12,13]. Therefore, developing methods to eliminate GICs is essential for effective GBM treatment.

Utilizing GICs and chemical libraries, we and others have identified inhibitors of dihydroorotate dehydrogenase (DHODH), a critical enzyme in the de novo pyrimidine biosynthetic pathway, as effective agents that selectively kill GICs while exhibiting lower toxicity to normal cells, including NSCs, neurons, and astrocytes [14,15]. DHODH inhibitors (DHODH-I) have also been shown to induce myeloid differentiation of acute myeloid leukemia and deprive stemness by exporting SOX2 from the nucleus in GICs [15,16]. Furthermore, administering a DHODH-I, such as brequinar (BRQ), prevents GIC tumorigenesis in immunodeficient mice, suggesting that DHODH-I is a promising chemical for GBM therapy.

While the use of DHODH-I holds promise for tumor eradication, the physical compression and inflammation induced by the tumor can compromise the surrounding functional neural cells, leading to impaired brain function in the affected region [17,18]. Regenerative medicine, particularly through the transplantation of NSCs and functional neural cells, presents a potential avenue for restoring these functions. However, given the substantial size of brain tumors in patients, it is unlikely that cell transplantation alone can achieve complete reconstruction of brain tissue and restoration of neural function. Emerging evidence suggests that GBM cells extensively integrate into the brain’s neuronal circuits to facilitate their proliferation and maintenance [19,20,21]. These insights raise the possibility that modulating the differentiation state of GBM cells, particularly GICs, may alter tumor–neural interactions and potentially preserve neural circuit integrity within the tumor-bearing brain.

In our study, we demonstrated that the sequential administration of a neuronal inducer and DHODH-I to mice with GIC brain tumors not only prompted the differentiation of GICs into neuron-like cells exhibiting synapse-associated structural features, but also eradicated undifferentiated GICs. This method preserved motor functions, which typically diminish during tumorigenesis, and significantly prolonged the survival of tumor-injected mice. Consequently, this strategy of preserving neural function within the tumor-bearing brain by normalizing GBM cells signifies a paradigm shift in GBM therapy and enhances the QOL for patients.

## 2. Materials and Methods

### 2.1. Animals and Chemical Reagents

All experiments involving mice were conducted according to the protocols approved by the Animal Care and Use Committee of Hokkaido University (Approval Code: 22-0079, Approval date: 4 July 2022). KSN/Slc nude mice, which were generated by introducing the *nu* gene into DDD/1 mice, were obtained from Sankyo Lab Service Corporation Japan (SLC Japan, Tokyo, Japan) and maintained in a specific pathogen-free (SPF) room during the experiments. When an animal is subjected to unbearable conditions, as evidenced by symptoms such as shivering and weight loss, it is crucial to evaluate the implementation of humane endpoints, including euthanasia, at a suitable time. Isoxazole 9 (ISX9, HY-12323) and brequinar (BRQ, 6196) were purchased from MedChemExpress (Monmouth Junction, NJ, USA) and Tocris Bioscience (Bristol, UK), respectively. Other chemicals and growth factors were purchased from Invitrogen (Carlsbad, CA, USA), Sigma-Aldrich (St. Louis, MO, USA), and PeproTech (Rocky Hill, NJ, USA), unless otherwise indicated.

### 2.2. Cell Culture

Human GIC lines (E6 and E16 that are pro-neural and mesenchymal types, respectively) were established from an independent GBM specimen and utilized in accordance with the research guidelines approved by the Ehime University Graduate School of Medicine and the Institute for Genetic Medicine, Hokkaido University. Cells were cultured in a medium composed of 50% DMEM/F12 (Sigma-Aldrich, D8062) supplemented with heparin (5 μg/mL; SIGMA, 9041-08-1), bFGF (20 ng/mL; PeproTech, 100-18B), and EGF (20 ng/mL; PeproTech, AF-100-15), hereafter referred to as NSC medium, combined with 50% DMEM (Nacalai Tesque, Kyoto, Japan, 16971-55) containing 10% fetal calf serum (FCS), as previously described [22]. Cell proliferation was evaluated using an MTT assay following established procedures [23]. GICs were treated with an anti-Mycoplasma removal agent (Wakenbtech Co., Ltd., Kyoto, Japan, MC-210) every six months.

### 2.3. RNA Sequencing Analysis

Total RNA was extracted from GICs cultured in the presence of either DMSO alone or ISX9 for three days using an RNeasy Mini Kit (QIAGEN, Hilden, Germany, 74104). RNA-seq libraries were prepared using the QuantSeq 3′mRNA-Seq Library Prep Kit for Illumina (FWD) <015.384> (Lexogen GmbH, Vienna, Austria), following the manufacturer’s instructions. Sequencing was performed using NextSeq500 (Illumina, San Diego, CA, USA). Sequence data were analyzed using bcl2fastq ver2.17 (Illumina) and StrandNGS ver4.0 (Strand Life Sciences Pvt Ltd., Bengaluru, India), according to the manufacturer’s guidelines. Expression profiles were analyzed using integrated differential expression and pathway analysis (iDEP, http://bioinformatics.sdstate.edu/idep/ accessed on 29 March 2022).

### 2.4. Immunocytochemical Analysis

Immunocytochemistry was conducted as previously described [24]. Briefly, cells were fixed in 2% paraformaldehyde prepared in PBS for 10 min at room temperature (RT), followed by blocking with buffer containing 5% FCS and 0.3% Triton X-100 in PBS for 30 min at RT. Samples were then incubated with primary antibodies (Abs) diluted in blocking buffer for 2 h at RT. After three washes with PBS, cells were incubated with appropriate secondary Abs together with Hoechst 33342 (1 μg/mL; Thermo Fisher Scientific, Waltham, MA, USA, H3570) for 1 h at RT. Following additional PBS washes, samples were mounted using Fluoromount (Sigma-Aldrich, F4680). Primary Abs used were as follows: mouse anti-βIII tubulin (1:100, R&D Systems, Minneapolis, MN, USA, MAB1195), mouse monoclonal anti-Nestin (1:200, BD Biosciences, San Jose, CA, USA, 611658), mouse anti-human synapsin I/II/III (1:1000, hSynapsin, Biolegend, San Diego, CA, USA, 16245), mouse monoclonal anti-Epithelial-V-like antigen 1 (1:200, EVA1, clone B2E5), chicken anti-βIII tubulin (1:5000, Novus Biologicals, Centennial, CO, USA, NB100-1612), chicken anti-microtubule associated protein 2 (1:5000, MAP2, Novus Biologicals, NB300-213), rabbit anti-GFAP (1:500, Dako, Glostrup, Denmark, N1506), and rabbit anti-Ki67 (1:250, ThermoFisher, MA5-14520). Signals were detected using Alexa488- or Alexa594-conjugated goat anti-mouse IgG (1:500, Thermo Fisher Scientific, A-11032), Alexa 488- or Alexa 594-conjugated goat anti-chicken IgY (1:500, Jackson ImmunoResearch, West Grove, PA, USA, 103-547-008), or Alexa 594-conjugated goat anti-rabbit IgG (1:500, Thermo Fisher Scientific, A10523). Fluorescence images were captured with an AxioImager A1 microscope (Zeiss, Oberkochen, Germany).

### 2.5. Intrathecal Injection

Chemical injections were performed via lumbar puncture into the subarachnoid space of the lumbar thecal sac. Conscious animals were manually restrained, and injections were performed free-hand at the posterior midline near the L4–L5 level (Supplementary Figure S1A,B), following a previously reported protocol [25].

### 2.6. In Vivo Toxicity Test

Considering the in vitro efficacies of BRQ and ISX9, in vivo toxicity tests were performed as follows: Ten microliters of two different concentration of BRQ, 0.02 or 0.1 mM, were injected once or twice per week intrathecally (Supplementary Figure S1C). Ten microliters of three different concentrations of ISX9 (0.1, 0.2, or 0.5 mM) were injected intrathecally twice a week (Supplementary Figure S1D). The survival of the injected mice was monitored for 40 days. Ultimately, we decided to inject 0.1 mM BRQ and 0.2 mM ISX9 twice a week for the in vivo experiments. It should be noted that we did not observe any toxicity in mice injected with either chemicals suspended in 10% DMSO or 10% DMSO alone.

### 2.7. Brain Tumorigenesis

Brain tumor formation was conducted according to a previously reported protocol [23]. Briefly, 1 × 10^5^ human GICs were resuspended in 5 μL of culture medium and stereotactically implanted into the brains of male nude mice (10–12 weeks old) under anesthesia using a combination of medetomidine, midazolam, and butorphanol. Injection coordinates were set at 2 mm anterior to the lambda, 2 mm lateral to the sagittal suture, and 4 mm deep. Fourteen days after transplantation, mice received intrathecal administration of 10 μL containing 0.1 mM BRQ, 0.2 mM ISX9, or 10% DMSO twice weekly for a total of four treatments.

To analyze the neuronal function of GIC-brain tumor cells, GICs were injected into the motor cortex at coordinates 1 mm forward from the bregma, 1.5 mm lateral from the sagittal suture, and 1.5 mm in depth. Fourteen days after GIC transplantation, 10 μL of 10% DMSO, 0.1 mM BRQ, or 0.2 mM ISX9 was intrathecally injected into the mice twice a week for two weeks. For sequential injection of ISX9 and BRQ, ISX9 was injected into the mice twice in the first week, followed by two injections of BRQ in the subsequent week.

### 2.8. Grip Strength Test

The grip strength test was performed to evaluate forelimb and hindlimb muscle strength using a Newton Meter (Nacalai Tesque, A05-4065) with a stainless wire mesh (16 × 10.8 cm), as shown in the Supplementary Video. Prior to the experiment, male nude mice aged 8–12 weeks were trained using a grip strength meter. Briefly, each mouse was gently held by the base of the tail and allowed to grasp the horizontal bar of the grip strength meter with both forelimbs and hindlimbs. Once the mouse secured its grip, the tail was gently pulled back along the horizontal plane until the mouse released its grip on the bar. The maximum force exerted before release was recorded in newtons (N) by the device. Each mouse underwent three consecutive trials with a 2 min inter-trial interval to avoid fatigue. The mean of the three grip strength values was calculated and used in subsequent analyses. All experiments were conducted at the same time of the day to control for circadian variations in muscle performance.

### 2.9. Immunohistochemical Analysis

Mouse brains were collected, embedded in Tissue-Tek OCT compound (Miles, Redwood City, CA, USA), and stored at −80 °C overnight. Coronal brain sections (14 μm thickness) were obtained from the cerebral cortex and subjected to hematoxylin and eosin (H&E) staining according to standard histological procedures. For immunohistochemical analysis, antigen retrieval was performed using HistoVT One (Nacalai Tesque, 06380-05) in accordance with the manufacturer’s protocol. The following Abs were used to detect antigens: mouse monoclonal anti-human mitochondria (1:200, hMito, Novus Biologicals, NBP2-32980-0.1 mg), mouse monoclonal anti-hSynapsin I/II/III as a pre-synapse marker (1:1000, Biolegend), mouse monoclonal anti-βIII tubulin (1:200, R&D Systems, MAB1195), chicken monoclonal anti-MAP2 (1:5000, Novus Bio), chick monoclonal anti-βIII tubulin (1:1000, Novus Bio, NB100-1612), rabbit polyclonal anti-cleaved Caspase 3 (1:1000, Casp3, Cell Signaling Technology, Danvers, MA, USA, 9661), rabbit polyclonal anti-MPZL2 (1:100, EVA1, Proteintech, Rosemont, IL, USA, 11787-1-AP), rabbit polyclonal anti-neurofilament M (1:100, NFM, Sigma-Aldrich, AB1987), and rabbit polyclonal anti-postsynaptic density protein 95 (1:500, PSD95, Proteintech) as a post-synapse marker. Secondary Abs included Alexa488- or Alexa594-conjugated goat anti-mouse IgG (1:500, ThermoFisher, A-11032), Alexa488- or Alexa594-conjugated goat anti-chicken IgY (1:500, Jackson ImmunoResearch, 103-547-008) and Alexa594-conjugated goat anti-rabbit IgG (1:500, ThermoFisher, A10523). Fluorescent images were captured using an AxioImager A1 microscope (Zeiss). Tumor area relative to total brain area and the proportion of marker-positive signals per section were quantified using ImageJ software (version 1.51j8; National Institutes of Health, Bethesda, MD, USA). Quantification results were also verified by visual assessment to ensure consistency with morphological observations.

### 2.10. Statistical Analysis

Statistical analyses were performed using *t*-test, one-way ANOVA, and log-rank (Mantel–Cox) test. Survival data were analyzed for significance using Kaplan–Meier methods with GraphPad Prism (version 9.0; GraphPad Software, San Diego, CA, USA, *p*-values were calculated using the log-rank test).

## 3. Results

### 3.1. Intrathecal Injection of BRQ Prevented GIC Tumorigenesis in the Brain

**Figure 1:**
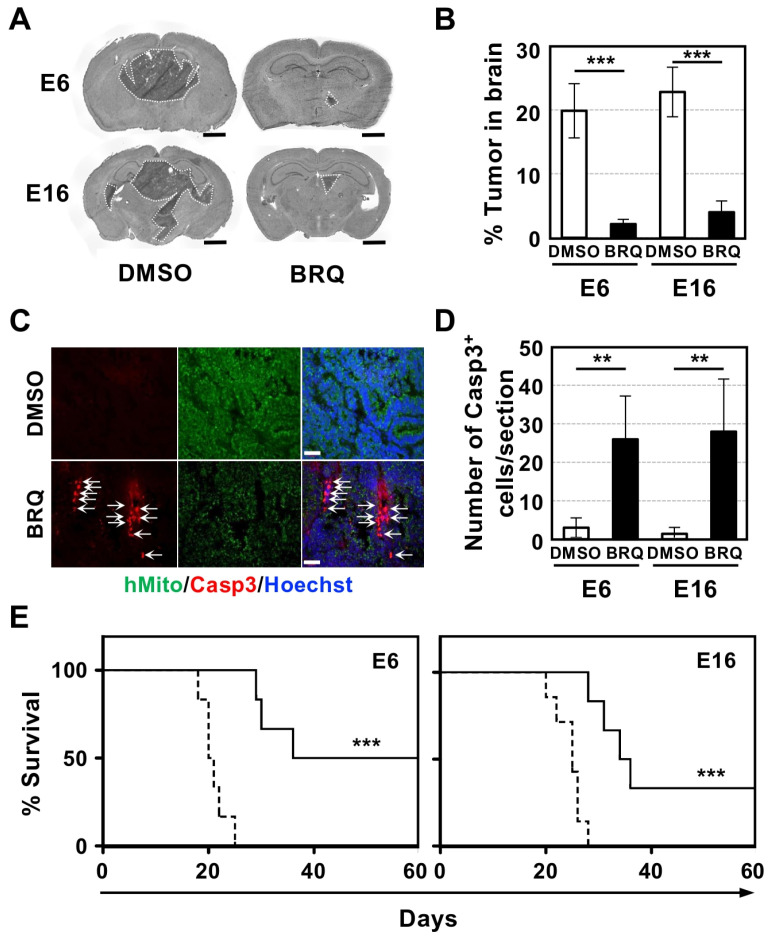
Intrathecal injection of BRQ prevented GIC tumorigenesis in brain and extended survival time of brain tumor-bearing mice. (**A**) H&E staining of coronal sections of GIC tumor-bearing brains, which were injected with DMSO or BRQ intrathecally. Tumors are surrounded by a white dotted line. Scale bar: 1 mm. (**B**) Quantitative data of tumor size in the brain corresponding to (**A**). (**C**) Representative images of immunoreactivity for hMito (green) and Casp3 (red) in E6 brain tumors in mice that were injected intrathecally with either DMSO or BRQ. Arrows show Casp3^+^ dead GICs. All nuclei were labeled with Hoechst33342 (Hoechst, blue). Scale bar: 50 μm. (**D**) Quantitative data of the number of Casp3^+^ cells per section of the GIC tumor-bearing brain corresponding to (**C**) (*n* = 5). (**E**) Survival curve of GIC tumor-bearing mice injected with DMSO (dashed line) or BRQ (solid line) (*n* = 6). Statistical significance was determined using the *t*-test (**B**,**D**) and log-rank test (**E**). Error bars indicate ± SD. ** *p* < 0.01, *** *p* < 0.001.

We previously demonstrated that BRQ effectively prevents tumorigenesis of subcutaneously transplanted GICs [15]. Given that BRQ cannot cross the blood–brain barrier (BBB), we examined whether intrathecal injection, a clinical method, could prevent GIC tumorigenesis in the brain [26]. Fourteen days after GIC transplantation into the brains of nude mice, we administered BRQ intrathecally twice a week for two weeks. Two days after the last BRQ injection, brain sections were prepared and tumorigenesis was analyzed using H&E staining. We observed significant reductions in tumorigenesis in BRQ-treated mice compared to that in the control (DMSO alone) group (Figure 1A). Tumor sizes in E6- and E16-transplanted brains were approximately 20% and 23%, respectively, while those in BRQ-treated brains were only 2% and 4%, respectively (Figure 1B).

To confirm that BRQ killed GICs in the tumors, we immunolabeled brain sections for human mitochondria (hMito) and the cell death marker cleaved-Caspase 3 (Casp3). We detected extensive Casp3^+^ signals in BRQ-treated GIC tumors, whereas few signals were observed in the control brains (Figure 1C). The percentage of Casp3^+^ cells in the E6 and E16 brain tumors treated with BRQ was approximately 26% and 28%, respectively, compared to 3% and 2% in the control (Figure 1D).

Survival analysis confirmed that BRQ injection significantly extended the survival time of GIC tumor-bearing mice (Figure 1E). The median survival of BRQ-treated tumor-bearing mice was 48 days for E6 and 35 days for E16, whereas control mice survived for 21 and 25 days, respectively. Collectively, these findings reveal the strong anti-GIC brain tumor activity of BRQ in vivo.

### 3.2. ISX9 Induced Neuronal Differentiation of GICs in Culture

Many compounds, including ISX9 and SB431542, have been shown to induce neurogenesis from NSCs, glioma cells, and reactive astrocytes [27,28]. We selected ISX9 as the most promising candidate because it not only induces neurogenesis by activating master genes, including *NeuroD1* and the myocyte-enhancer family of proteins (*MEF2*), but also crosses the BBB [27,29,30], suggesting that ISX9 is a candidate for inducing GICs to differentiate into neurons.

**Figure 2:**
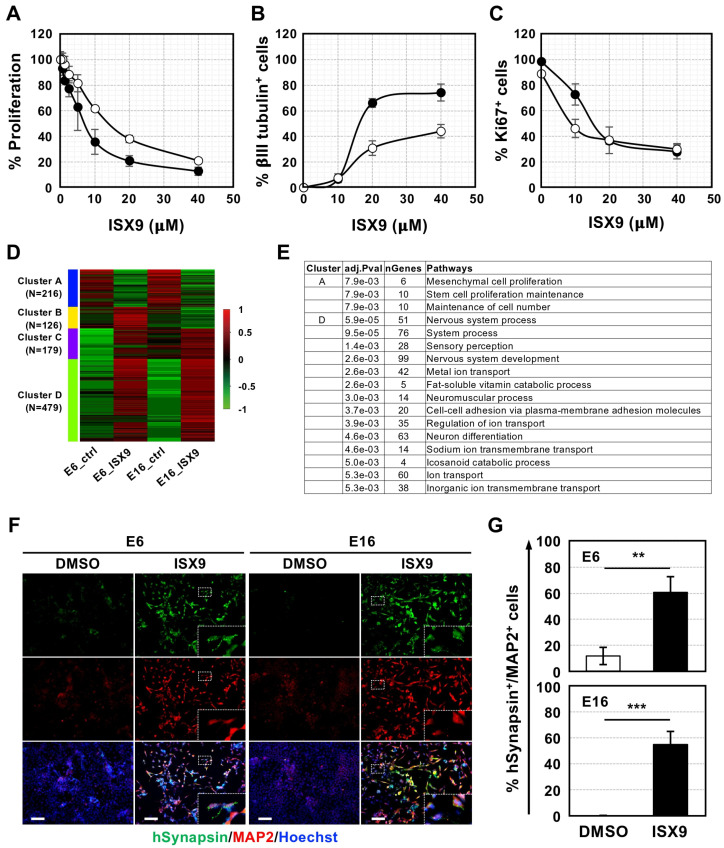
ISX9 induced GICs to differentiate into neurons. (**A**) Proliferation of GICs, E6 (closed circle) and E16 (open circle), cultured in the presence of various concentrations of ISX9, was determined using the MTT assay. (**B**,**C**) Dose-dependent ISX9 effects on βIII tubulin (**B**) and Ki67 (**C**) in GICs, E6 (closed circle), and E16 (open circle). (**D**) Heat map with hierarchical clustering of the top 1000 genes in GICs, E6, and E16, cultured with DMSO or ISX9 (30 μM) for 3 days. (**E**) Enriched pathways for clusters A and D. (**F**) Immunoreactivity of hGICs cultured in the presence of ISX9 (30 μM) for 3 days, for hSynapsin (green) and MAP2 (red). The higher magnification images are shown on the bottom right of each figure surrounded by white dotted lines. All nuclei are labeled with Hoechst33342 (Hoechst, blue). Scale bar: 20 μm. (**G**) Quantitative data of (**F**). ** *p* < 0.01, *** *p* < 0.001. Statistical significance was determined using the *t*-test. Error bars indicate ±SD.

To investigate whether ISX9 induces neuronal differentiation in GICs, we cultured the cells with various concentrations of ISX9 and observed that its addition inhibited GIC proliferation. To quantify the anti-proliferative effect of ISX9, we performed an MTT assay. As shown in Figure 2A, ISX9 inhibited GIC proliferation in a dose-dependent manner, with the Half Maximal Inhibitory Concentration (IC50) values of approximately 7 µM for E6 and 14 µM for E16.

We then immunolabeled ISX9-treated GICs with the neuronal marker βIII tubulin and the proliferation marker Ki67. In the presence of 40 µM ISX9, approximately 74% of E6 cells and 44% of E16 cells were positive for βIII tubulin, while Ki67^+^ proliferating GICs decreased to less than 30% (Figure 2B,C; Supplementary Figure S2A,B).

Additionally, ISX9 treatment reduced the expression of Nestin, which is not only a well-known NSC marker but is also involved in tumorigenicity [31], whereas the expression of the astrocyte marker GFAP remained unchanged (Supplementary Figure S2A,B). Together, these results indicate that ISX9 induces neuronal differentiation accompanied by cell cycle arrest in GICs.

To further assess the extent of ISX9-induced neuronal differentiation in GICs, we conducted RNA-seq analysis. Comparing the expression profiles of control GICs with those of ISX9-treated cells, we found that 3646 and 3639 genes were upregulated in ISX9-treated E6 and E16, respectively, whereas 2143 and 2398 genes were downregulated in these cells.

Hierarchical clustering of the top 1000 genes revealed the upregulation of pathways related to neuronal development, nervous system development and processes, cell adhesion, and transmembrane transport, alongside the downregulation of pathways associated with stem cell proliferation. This indicates that ISX9 induced neuronal differentiation in GICs, accompanied by genome-wide changes in gene expression (Figure 2D,E, Supplementary Figure S3A).

RNA-seq analysis further revealed the upregulation of mature neuronal marker genes, including sodium channels, synapsin, glutamate receptors, and synaptoporin, along with the downregulation of stem cell-related genes (Supplementary Figure S3B,C). Among these mature neuronal genes, we focused on the expression of the presynaptic marker synapsin, which is essential for forming functional neuronal circuits, in ISX9-treated GICs using immunocytochemical analysis. As shown in Figure 2F, ISX9 significantly increased human synapsin (hSynapsin) expression in GICs, with approximately 60% of E6 and 58% of E16 being double-positive for hSynapsin and MAP2 (Figure 2G). In contrast, only approximately 12% of E6 and none of E16 were double-positive when cultured with DMSO alone. These data indicate that ISX9 can induce GIC differentiation into neuron-like cells expressing synaptic markers and structural features associated with synapse formation.

### 3.3. ISX9 Prevented GIC Tumorigenesis and Induced Their Neuronal Differentiation in the Brain

**Figure 3:**
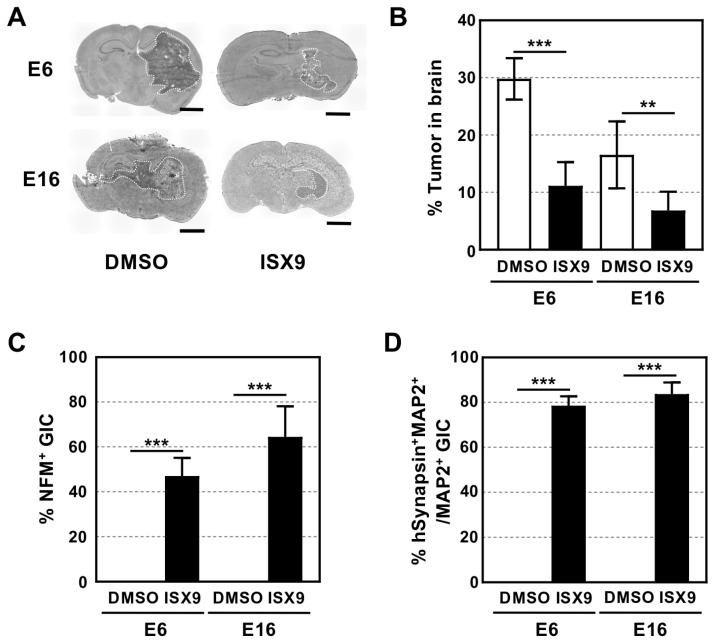
Intrathecal injection of ISX9 induced GIC-derived brain tumor cells to differentiate into synapse-forming neurons. (**A**) H&E staining of coronal sections of GIC tumor-bearing brains, which were injected intrathecally with DMSO or ISX9. Tumors were surrounded by white dotted line. Scale bar: 1 mm. (**B**) Quantitative data of tumor size in brain corresponding of (**A**). (**C**) Quantitative data of NFM+ GICs per section of GIC tumor-bearing brain injected with DMSO or ISX9. (**D**) Quantitative data of hSynapsin^+^ MAP2^+^/MAP2^+^ GICs per section of GIC tumor-bearing brain injected with DMSO or ISX9 (*n* = 6). Statistical significance was determined using the *t*-test. Error bars indicate ±SD. ** *p* < 0.01, *** *p* < 0.001.

Next, we examined whether intrathecal injection of ISX9 induced GIC differentiation into neurons in the brain. Two weeks after GIC transplantation, we intrathecally injected ISX9 (0.2 mM) twice a week for two weeks. Four weeks after transplantation, brain sections were prepared and stained with H&E. As shown in Figure 3A, ISX9 significantly prevented GIC tumorigenesis in the brain tissue. The tumor sizes of E6 and E16 in the brains were approximately 30% and 17%, respectively, whereas those in ISX9-treated brains were reduced to 11% and 7%, respectively (Figure 3B). To validate whether ISX9 induced neuronal differentiation in GIC tumor cells, we immunolabeled brain sections with hMito and the neuronal marker neurofilament middle size (NFM). We observed a large number of NFM^+^ GIC-derived neurons (arrows) in ISX9-treated brain tumors, whereas no NFM^+^ human cells were detected in the control tumors (Supplementary Figure S4A). Approximately 47% of E6 and 65% of E16 tumor cells were positive for NFM (Figure 3C). Additionally, GIC-derived MAP2^+^ neurons were also positive for hSynapsin, with approximately 79% and 84% of MAP2^+^ E6 and E16 tumor cells being immunolabeled for hSynapsin, respectively (Figure 3D, Supplementary Figure S4B). It should be noted that many hSynapsin^+^ GIC-derived cells invaded the mouse brain (arrowheads). We further found an increased number of postsynaptic factor PSD95^+^ GIC-derived cells in the tumor (arrows) and host mouse brain (arrowheads) (Supplementary Figure S4C). Together, these data suggest that ISX9 induces neuronal differentiation of GICs and promotes the formation of synapse-like structures between GIC-derived cells and surrounding host brain tissue. These structural changes may contribute to remodeling of the tumor–neural interface.

### 3.4. Sequential Treatment of GIC from ISX9 to BRQ Is the Optimal Method for Inducing Large Number of Neurons and Eliminating Undifferentiated GICs

To achieve the goals of this study, we investigated the optimal method for generating large quantities of GIC-derived mature neurons while eliminating undifferentiated GICs. We examined the following three strategies: First, we administered both chemicals simultaneously. Second, we applied ISX9 first, and then switched to BRQ. Third, we began treatment with BRQ and subsequently switched to ISX9. We cultured GICs under these conditions and immunolabeled for two neuronal markers, βIII tubulin and MAP2, along with a GIC marker EVA1 that is involved in the malignancy and is a potential target for the therapy [32,33], and Ki67 as a proliferation marker. We also counted the number of surviving cells after implementing these strategies.

**Figure 4:**
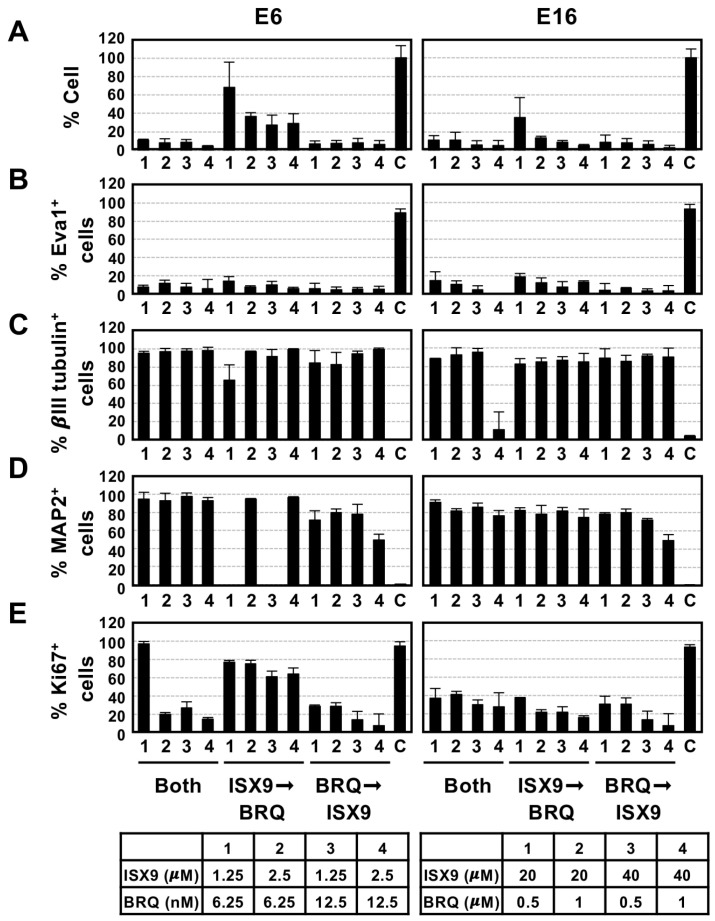
Examination of optimal method that induces neurons from as many GICs as possible and eliminates undifferentiated GICs. GICs were cultured under three culture conditions, both ISX9 and BRQ simultaneously, ISX9 followed by BRQ, and BRQ followed by ISX9, with either the maximum concentration that induced neuronal differentiation or half the concentration, as shown in Figure 2 and previously [15]. The surviving cells were counted (**A**) and immunolabeled for the GIC marker EVA1 (**B**), two neuronal markers, βIII tubulin (**C**) and MAP2 (**D**), and the proliferation marker Ki67 (**E**). The table shows the concentrations of the chemicals used in each experiment. The arrows indicate the sequential chemical treatments. Error bars indicate ±SD. Statistical significance was determined using one-way ANOVA. Error bars indicate ±SD.

We observed that over 90% of GICs died when treated with both chemicals simultaneously or when BRQ was administered, followed by ISX9. In contrast, a significant number of cells survived when cultured with ISX9 followed by BRQ (Figure 4A). The surviving cells were negative for EVA1 (positivity is less than 20%), while approximately 80% of the cells were positive for both neuronal markers, except for MAP2 immunoreactivity in E6 cultured at a low concentration of ISX9 followed by BRQ (Figure 4B–D; Supplementary Figure S5). Additionally, over 60% of E6 were Ki67^+^ in the ISX9 followed by BRQ conditions, whereas less than 20% of E6 and 30% of E16 cultured under other conditions were positive for Ki67 (Figure 4E; Supplementary Figure S5C,D). Together, these data suggest that sequential treatment with ISX9 followed by BRQ efficiently promotes neuronal differentiation-like conversion while reducing the population of undifferentiated tumor cells.

### 3.5. Sequential Administration of ISX9 and BRQ Maintained Grip Strength in GIC Brain Tumor-Bearing Mice and Extended Their Survival Time

**Figure 5:**
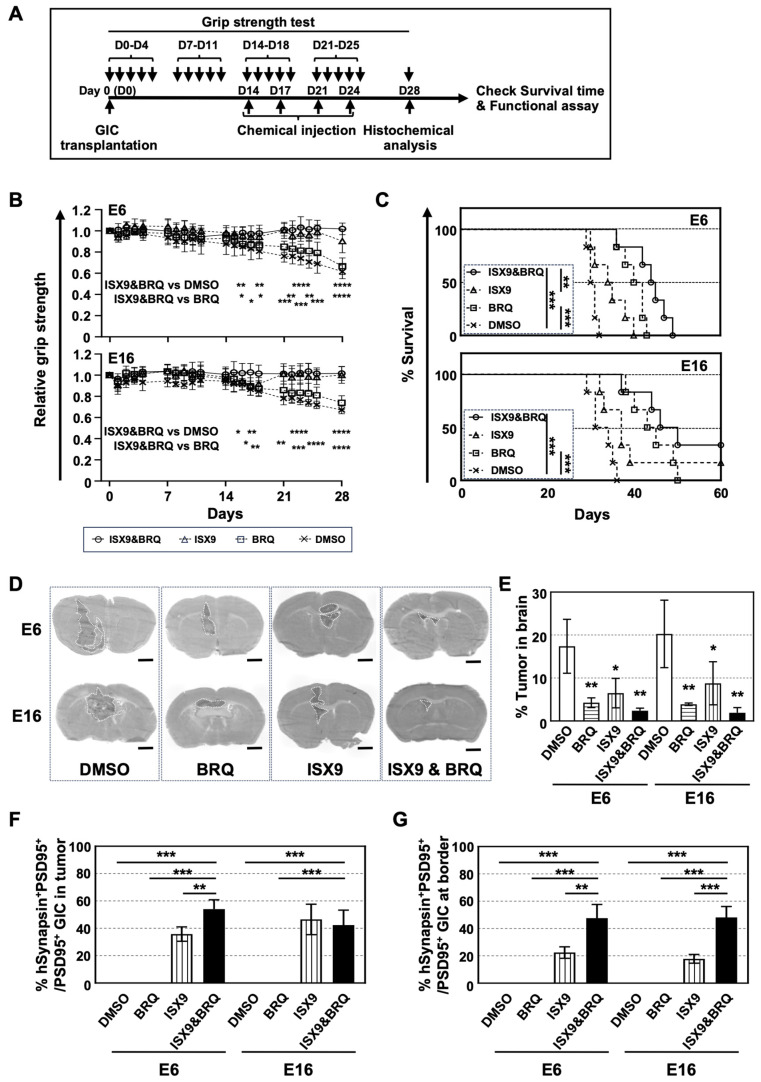
Sequential administration of ISX9 and BRQ prevented GIC tumorigenesis in brain while maintaining grip strength in the tumor-bearing mice. (**A**) Experimental procedures for examining the effects of ISX9 and BRQ on GIC brain tumors. (**B**) Grip strength of GIC tumor-bearing mice injected with DMSO, BRQ, ISX9, and ISX9&BRQ. Statistical significance was observed for DMSO compared to ISX9 and DMSO compared to ISX9&BRQ. (**C**) Survival curve of GIC tumor-bearing mice injected with DMSO, BRQ, ISX9, or ISX9&BRQ. The following symbols and lines were used in panels (**B**,**C**): DMSO (cross, dashed line), BRQ (square, dashed line), ISX9 (triangle, dashed line), and ISX9&BRQ (circle, solid line) (*n* = 6). (**D**) H&E staining of coronal sections of GIC tumor-bearing brains, which were injected with DMSO, BRQ, ISX9, or ISX9&BRQ intrathecally. Tumors were surrounded by white dotted line. Scale bar: 1 mm. (**E**) Quantitative data of tumor size in brain corresponding of (**D**). (**F**,**G**) Quantitative data of hSynapsin+ PSD95+/PSD95+ GICs in the tumor (**F**) or at the border (**G**) per section of GIC tumor-bearing brain intrathecally injected with DMSO (white column), BRQ (horizontal line column), ISX9 (vertical line column), or ISX9&BRQ (black column) (*n* = 3). Statistical significance was determined using one-way analysis of variance (ANOVA) (**B**,**E**–**G**) and log-rank test (**C**). Error bars indicate ± SD. * *p* < 0.05, ** *p* < 0.01, *** *p* < 0.001, **** *p* < 0.0001.

To investigate whether the optimal method identified in vitro contributes to regenerating the GIC tumor-bearing brain, we injected GICs into the motor cortex and measured the grip strength of mice treated with chemicals, following the schedule shown in Figure 5A. We found that both the injection of ISX9 alone (triangles) and the sequential injection of ISX9 and BRQ (circles) maintained grip strength in GIC tumor-bearing mice, whereas grip strength decreased over time in mice injected with either BRQ (squares) or DMSO alone (crosses) (Figure 5B).

All DMSO (crosses)-injected mice transplanted with E6 and E16 died by days 32 and 36, respectively, with median survival times of 30 and 31 days. In contrast, administration of either BRQ (squares) or ISX9 (triangles) alone siognificantly extended the survival time of GIC brain-tumor-bearing mice (Figure 5C). The median survival times of tumor-bearing mice injected with BRQ were 41 days for E6 and 44 days for E16, whereas those injected with ISX9 were 35 and 37 days. Sequential injection of ISX9 and BRQ (circles) further extended the median survival time of tumor-bearing mice to 45 days for E6 and 48 days for E16. Together, these data demonstrate that the sequential administration of ISX9 and BRQ not only maintained motor neuronal activity in GIC brain tumor-bearing mice but also prolonged their survival.

### 3.6. Sequential Administration of ISX9 and BRQ Prevented GIC Tumorigenesis and Induced GIC Differentiation into Neurons That Formed Synapse-like Structure Contacting with Mouse Neurons

To verify the therapeutic effects of ISX9 and BRQ against GIC brain tumors, we prepared brain sections 28 days after GIC transplantation and stained the sections with H&E (Figure 5D). We observed that the administration of chemicals in all cases prevented GIC tumorigenesis, with the combination of ISX9 and BRQ showing the greatest effect on tumorigenesis. The percentage of E6 tumors in the brains injected with DMSO, BRQ, ISX9, and the combination were approximately 17%, 4%, 6%, and 2%, respectively. Similarly, the percentage of E16 tumors in the brains injected with DMSO, BRQ, ISX9, and the combination was approximately 20%, 4%, 9%, and 2%, respectively (Figure 5E).

To further analyze the anti-tumorigenic effects of the chemicals, we immunolabeled the sections for βIII tubulin, EVA1, and hMito (Supplementary Figure S6A). We found that injection of BRQ alone inhibited GIC tumorigenesis; however, the remaining tumor cells were EVA1-positive. Injection of ISX9 alone induced many βIII tubulin^+^ cells in the tumor, but many of the tumor cells were positive for EVA1. In contrast, the sequential administration of the two chemicals not only increased the number of βIII tubulin+ cells but also enhanced their signal intensity. Notably, this combination decreased the EVA1 signal in the tumor, although it did not completely eliminate EVA1^+^ cells (arrows in Supplementary Figure S6A). These data reveal that the sequential administration of the two chemicals not only converted GICs into neurons but also effectively reduced the number of EVA1^+^ cells.

The sequential administration of ISX9 and BRQ maintained grip strength in GIC-transplanted mice (Figure 5B). We investigated whether GIC-derived neurons were integrated into the neuronal circuit of the mouse brain by forming synapses with tumor-surrounding mouse neurons. We immunolabeled the sections for hSynapsin and a postsynaptic protein PSD95 and found an increased number of synapses that were positive for both markers in the tumor (arrowheads) and at the border between the tumor and the host mouse brain (arrows) following injection of either ISX9 alone or the combination (Supplementary Figure S6B). Approximately 36% and 47% of hSynapsin^+^ E6- and E16-derived neurons co-localized with PSD95 in the tumors of ISX9-injected mice. Similarly, 54% and 42% of hSynapsin^+^ E6- and E16-derived neurons co-localized with PSD95 signals in the tumor when both agents were injected sequentially (Figure 5F). At the border, we observed that 22% and 26% of hSynapsin^+^ E6- and E16-derived neurons co-localized with PSD95 in the ISX9-injected mice. When the two agents were injected sequentially, 48% and 34% of hSynapsin^+^ E6- and E16-derived neurons colocalized with the PSD95 signal (Figure 5G). These findings indicate increased formation of structural synaptic specializations between GIC-derived cells and host neurons following ISX9 treatment or sequential ISX9 and BRQ administration.

To further verify whether GIC-derived neurons form synapses with host mouse neurons, we immunolabeled the sections with MAP2, PSD95, and hMito. As shown in Supplementary Figure S6C, we observed close structural apposition between MAP2^+^ hMito^+^ GICs and MAP2^+^ hMito^−^ mouse neurons in brains exposed to ISX9 alone or in combination. Together with data that either ISX9 or the combination maintains grip strength in GIC brain tumor-bearing mice, these results suggest that GIC-derived neuron-like cells establish structural synaptic contacts with host mouse neurons and contribute to preservation of neuronal circuit integrity disrupted by tumorigenesis.

## 4. Discussion

Substantial evidence demonstrates that DHODH is a crucial target for various types of cancer, including melanoma, acute myeloid leukemia, breast cancer, pancreatic cancer, and GBM [14,15,34,35]. The administration of either DHODH-I alone or a combination of DHODH-I with conventional anti-cancer drugs has been shown to eradicate GBM cells in mice while being less toxic to non-cancer cells, including tissue-specific stem cells [14,15]. Therefore, it was crucial to determine how to deliver the inhibitor into the brain, as druggable DHODH-I, such as BRQ, cannot pass the BBB. We demonstrated that intrathecal injection, a clinically used method, efficiently delivered the compound into the mouse brain. Since the number of doses is limited to avoid causing damage to the spinal cord, it is still necessary to explore improved administration methods, including intracranial injection and nasal administration.

Our research demonstrated that ISX9 induced the expression of genes associated with neuronal differentiation and nervous system development in GICs and enhanced synapse formation in the tumor-bearing brain. Additionally, ISX9 preserved the grip strength of tumor-bearing mice, although its effectiveness diminished as tumor growth progressed. This preservation of grip strength can be partly attributed to the increased expression of several genes involved in synapse formation and function in ISX9-exposed GICs: *Glypican 5*, which regulates the maturation and stabilization of synaptic connections [36]; *CHRM3*, which participates in synapse formation with cholinergic neurons [37]; and *SLC6A1*, which removes GABA from the synaptic cleft and returns it to the presynaptic terminal [38]. However, using patch-clamp recordings, we could not detect stable action potentials in GIC-derived neurons in the brain, suggesting that GIC-derived neuron-like cells may not have achieved full electrophysiological maturation or stable functional synaptic transmission within the experimental timeframe, even though GBM cells have been shown to be integrated extensively into the host neuronal network [18,19,20]. One possible reason is that it takes a long time for functioning GIC-derived neurons to integrate into the resident neuronal network, as it has been shown to take over many months to more than a year post-implantation for human iPS cell-derived neurons transplanted into rodent brains to act as functional neurons with electrophysiological properties [39]. It was also indicated that structural synaptic connections between grafted and host neurons can be observed at early times, but therapeutically meaningful functional integration is thought to require prolonged periods [40], suggesting that early synapse formation does not equate to mature electrophysiological functionality.

Nonetheless, why could the administration of either ISX9 alone or the combination maintain the grip strength in GIC brain tumor-bearing mice? Extensive literature has demonstrated that glioma cells disrupt neuronal excitability and network integrity through multiple mechanisms, including altered glutamatergic and GABAergic signaling, ionic imbalance, and degradation of perineuronal nets, ultimately leading to neuronal hypoactivity or network silencing [41]. These circuit-level dysfunctions may underlie many motor and cognitive deficits observed in patients with glioma [42]. Together with our data, these findings suggest that neuronal differentiation of GICs may partially alleviate tumor-induced disruption of neuronal network activity, thereby contributing to maintenance of motor function.

The molecular mechanism underlying ISX9-dependent neuronal differentiation of NSCs relies on the induction of the neuronal transcription factors MEF2 and NeuroD [28,43]; however, we could not detect the induction of either transcription factor in ISX9-treated GICs. We also compared the genes regulated by the overexpression of MEF2 in NSCs or NeuroD in astrocytes with those in ISX9-exposed GICs. Three genes, *MAP2*, *NCAM1*, and *GFRA1*, were upregulated in both MEF2-overexpressing NSCs and ISX9-exposed GICs, whereas *SOX21* was downregulated in these cells [44]. Similarly, three genes, *DCX*, *RELN*, and *SNAP25*, were upregulated in both NeuroD-overexpressing NSCs and ISX9-exposed GICs, whereas two genes, *TNC* and *MYC*, were downregulated in these cells [45]. These results indicate that the effects of ISX9 vary significantly depending on the target cell type. Understanding the mechanisms underlying these transcriptional differences between GICs and normal neural cells may lead to the identification of novel molecular pathways involved in the neuronal differentiation of GICs.

BRQ-mediated DHODH inhibition robustly reduced tumor burden and extended survival but failed to preserve grip strength, reinforcing the notion that tumor shrinkage alone is insufficient to reverse the neurological dysfunction. The complementary effects observed with sequential ISX9 and BRQ administration further support a model in which differentiation-locked state induction and cytotoxic or cytostatic tumor control target distinct but synergistic aspects of glioma pathology. Together, these findings highlight the importance of therapeutically addressing both tumor burden and tumor–neural interface dysfunction to achieve meaningful functional benefits in glioblastoma.

## 5. Conclusions

Our strategy of combining the neuronal differentiation of GICs with the eradication of undifferentiated GICs represents a significant advancement in GBM therapy. Future studies should aim to refine this approach with additional methods, including the long-term maintenance of GIC-derived neurons and the induction of specific neuronal functions in the tumor-developing area, for clinical application.

## Data Availability

The original data presented in the study are openly available the Gene Expression Omnibus repository (accession number GSE325091).

## Supplementary Materials

The following supporting information can be downloaded at https://www.mdpi.com/article/10.3390/cells15060539/s1.

## Abbreviations

QOL	quality of life
GBM	Glioblastoma
GIC	GBM-initiating cells
NSC	neural stem cell
DHODH	dihydroorotate dehydrogenase
DHODH-I	DHODH inhibitor
BRQ	brequinar
ISX9	Isoxazole 9
Ab	antibody
BBB	blood–brain barrier
hMito	human mitochondria
Casp3	cleaved-Caspase 3
IC50	the Half Maximal Inhibitory Concentration
EVA1	epithelial-V-like antigen 1
NFM	neurofilament middle size
PSD95	postsynaptic density protein 95
