# Supplementary material for: Neuronal Differentiation of GBM-Initiating Cells Combined with Elimination of Undifferentiated Cells Preserves Motor Function
Source: Cells. 2026 Mar 18;15(6):539. doi: 10.3390/cells15060539 (PMC13026033; doi:10.3390/cells15060539)
Supplement: Supplementary file 1 [file cells-15-00539-s001.zip › cells-4180682-Supplementary information.pdf]

Supplementary information

Supplementary figure legends

Supplementary Figure S1. In vivo toxicity of BRQ and ISX9 via intrathecal injection

A, Schematic representation of the chemical injection site. Ten microliters of Fast green dye (1 % in PBS, blue) were injected at the posterior midline site at the lumbar (L) 4/5 level of the mouse. B, Distribution of the dye (blue) in the brain (upper images) and spinal cord (lower images) 30 min after injection. The right images of each tissue show coronal (upper) and rostrocaudal (lower) sections. The dye was successfully detected in both the brain and the spine. C, Kaplan-Meier curves of nude mice injected with BRQ once or twice a week. Two concentrations of BRQ, 0.02 (black) or 0.1 (red) mM, were administered either once (dashed line) or twice (solid line) a week for twoweeks. D, Kaplan-Meier curves for nude mice injected with various concentrations of ISX9:0.1 (black), 0.2 (blue), or 0.5 mM (red), administered twice a week for 2 weeks.

Supplementary Figure S2. Dose-dependent ISX9 effects on GICs

GICs, E6 and E16, were cultured with varying concentrations of ISX9 (0, 10, 20, or 40 μM) for 3 days. The cells were immunolabeled for either Nestin (green) and Ki67 (red) **(A)** or for βIII tubulin (green) and GFAP (red) **(B)**. All nuclei were labeled with Hoechst33342 (Hoechst, blue). Scale: 50 μm.

Supplementary Figure S3. RNA-seq analysis of ISX9-treated GICs

A, Heat map of the top 1000 genes in GICs, E6, and E16, cultured with DMSO (ctrl) or ISX9 (30 μM) for 3 days. **B,** The top 40 neuronal genes that were upregulated in ISX9-treated GICs compared to DMSO-treated GICs. **C,** The top 25 stem cell-related genes that were downregulated in ISX9-treated GICs compared to DMSO-treated GICs.

Supplementary Figure S4. Induced neuronal differentiation of GIC brain tumor cells via intrathecal injection of ISX9

GIC brain tumors obtained from tumor-bearing mice intrathecally injected with either DMSO or ISX9 were immunolabeled for hMito (green) and NFM (red) **(A)**, hSynapsin (green) and MAP2 (red) **(B)**, or hMito (red), MAP2 (green), and PSD95 (purple) **(C)**. Higher-magnification images are presented on the right of each figure, outlined by white dotted lines in the left images. Insets show GIC-derived postsynaptic neurons. Arrows indicate NFM^{+} and $PSD95^{+}$neurons in the tumors (**A** and **C**, respectively). Arrowheads indicate the synapsin + and PSD95 + GIC-derived neurons that invaded the mouse brain (**B** and **C**, respectively). All nuclei were labeled with Hoechst33342 (Hoechst, blue). Scale: 50 μm.

Supplementary Figure S5. Optimal strategy for targeting GICs in tumor-bearing brain regeneration

GICs, E6 **(A, C)** and E16 **(B, D)**, were cultured with BRQ, ISX9, or both for 2 days. The medium was subsequently switched to another chemical or maintained in both for another 2 days. The cells were immunolabeled with βIII tubulin (green) and EVA1 (red) or MAP2 (green) and Ki67 (red). All nuclei were labeled with Hoechst33342 (Hoechst, blue). Scale bar: 50 μm.

Supplementary Figure S6. Enhanced synapse formation in GIC brain tumor cells following sequential injection of ISX9 and BRQ

GIC brain tumor-bearing mice received intrathecal injections of DMSO, BRQ, ISX9, or a sequential injection of BRQ and ISX9. The brain sections were immunolabeled with the following markers: **A,** βIII tubulin (green), hMito (red), and EVA1 (purple); **B,** hSynapsin (green) and PSD95 (red); **C,** hMito (green), MAP2 (red), and PSD95 (purple). Arrowheads and arrows indicate GIC-derived neurons in the tumor and at the border, respectively. Asterisks denote GIC-derived neurons that invade the mouse brain. Stars in the insets illustrate the connection between GIC-derived neurons and mouse postsynaptic neurons. All nuclei were labeled with Hoechst33342 (Hoechst, blue). Scale bar: 50 μm and 25 μm (insets).

Supplementary video legend

Strategy for examining grip strength in brain tumor-bearing mice
