# Supplementary figures and images for: Neuronal Differentiation of GBM-Initiating Cells Combined with Elimination of Undifferentiated Cells Preserves Motor Function
Source: Cells. 2026 Mar 18;15(6):539. doi: 10.3390/cells15060539 (PMC13026033; doi:10.3390/cells15060539)

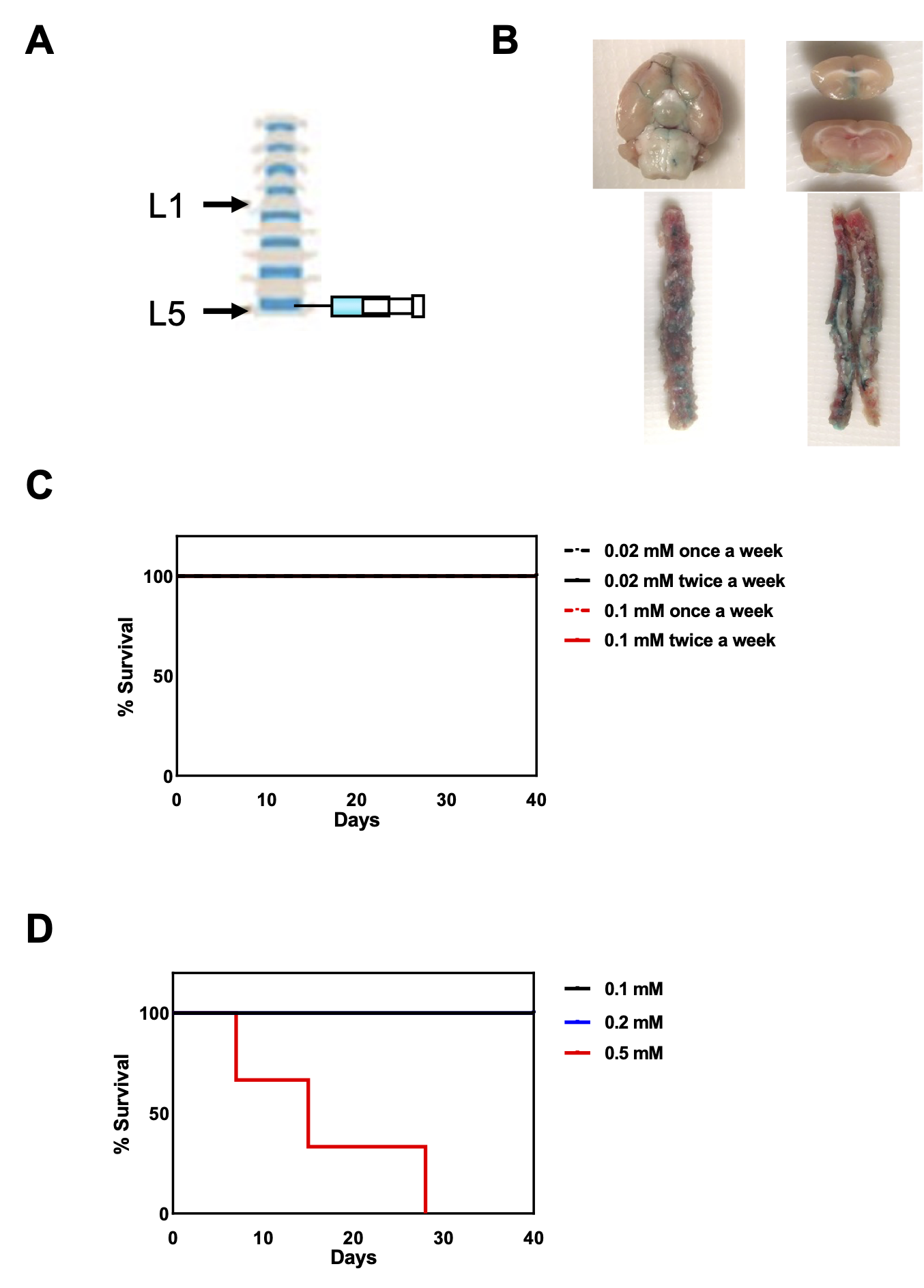

Supplement: Supplementary file 1 [file cells-15-00539-s001.zip › cells-4180682-Supplementary Figure S1.tiff]

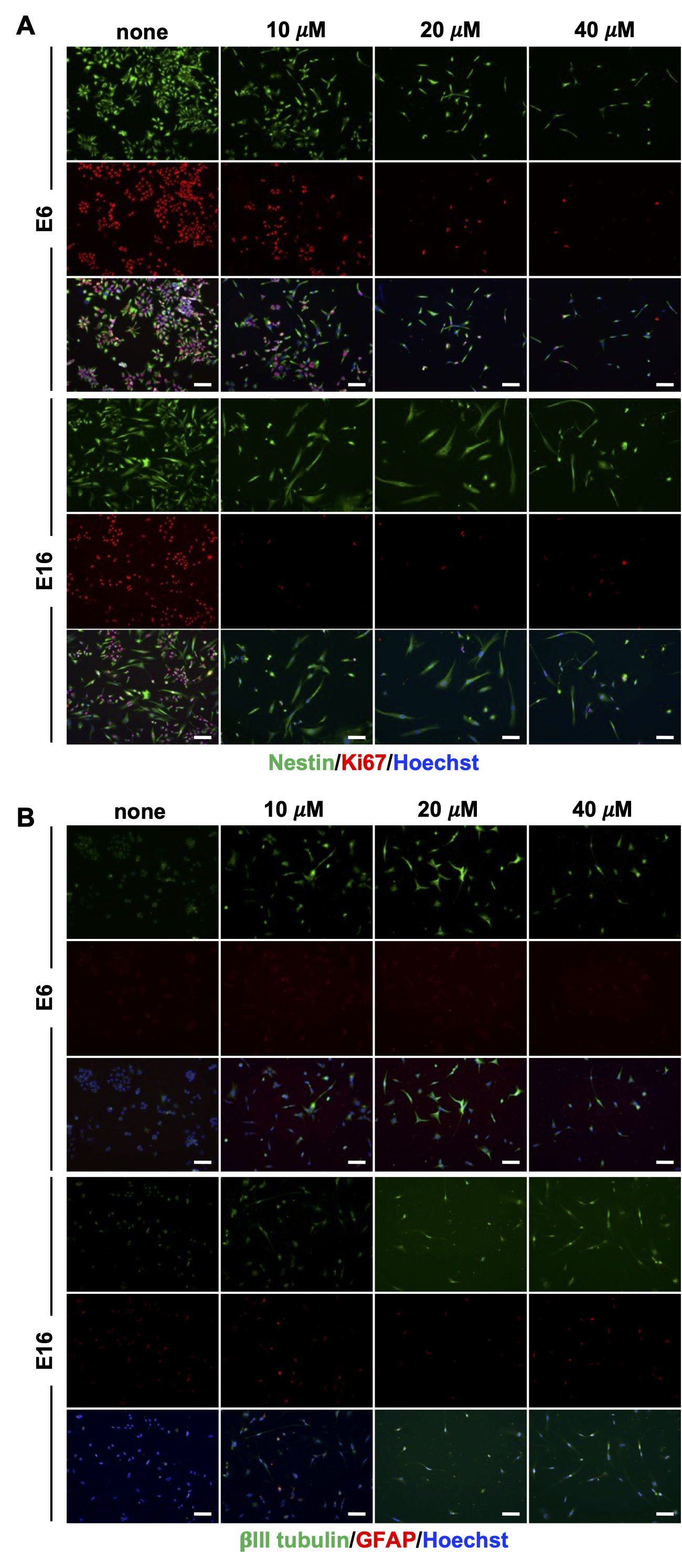

Supplement: Supplementary file 1 [file cells-15-00539-s001.zip › cells-4180682-Supplementary Figure S2.tiff]

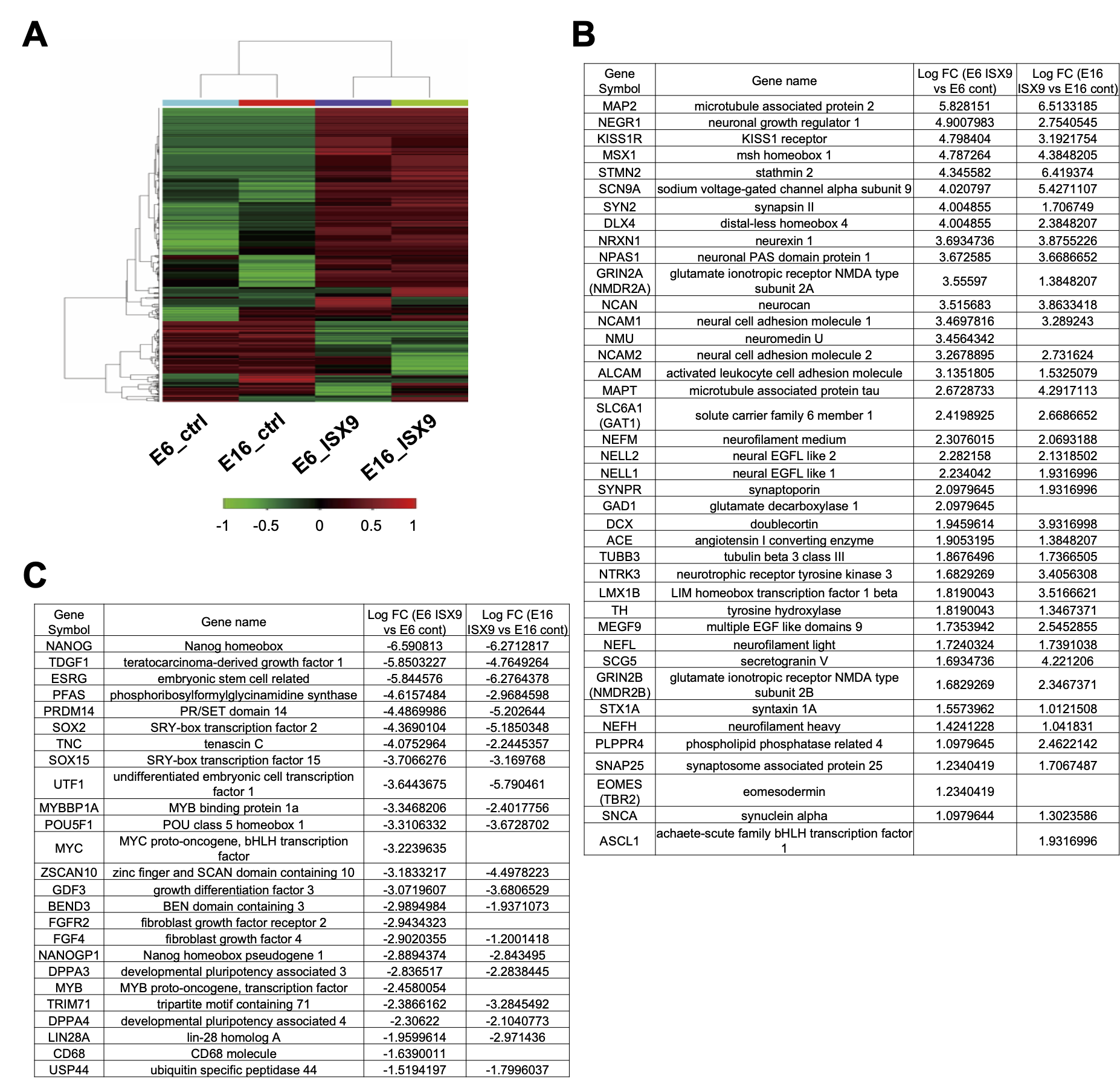

Supplement: Supplementary file 1 [file cells-15-00539-s001.zip › cells-4180682-Supplementary Figure S3.tiff]

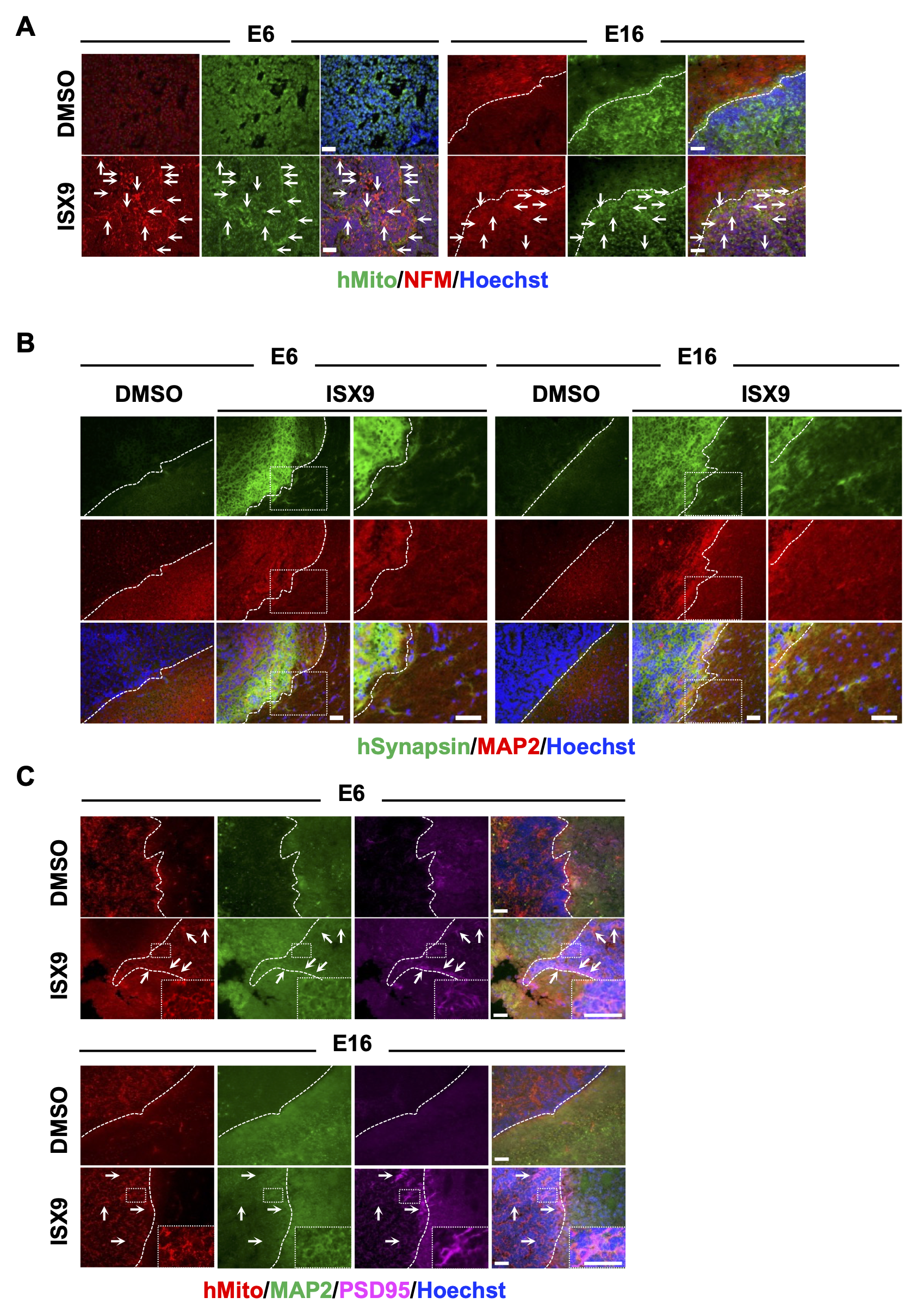

Supplement: Supplementary file 1 [file cells-15-00539-s001.zip › cells-4180682-Supplementary FIgure S4.tiff]

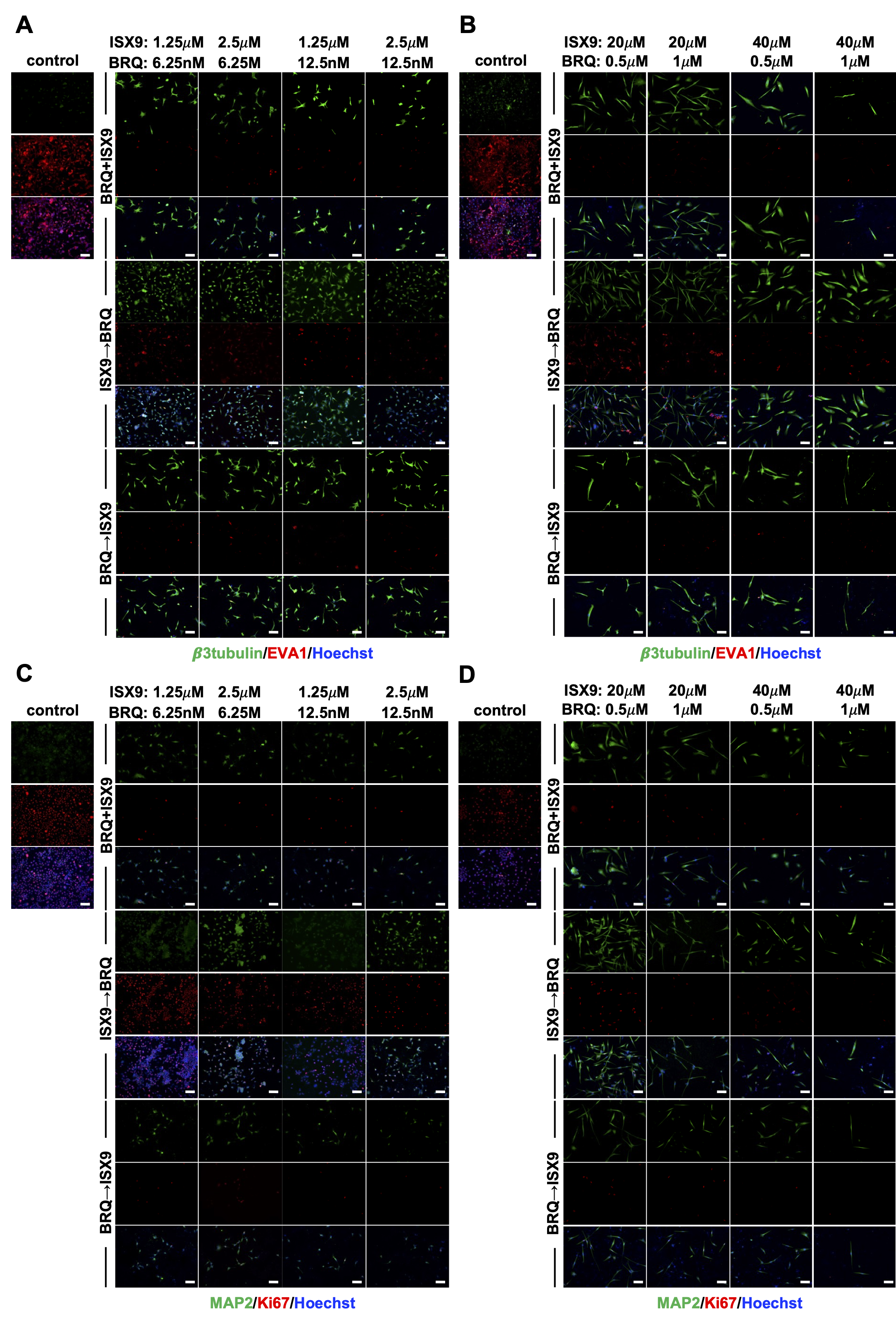

Supplement: Supplementary file 1 [file cells-15-00539-s001.zip › cells-4180682-Supplementary Figure S5.tiff]

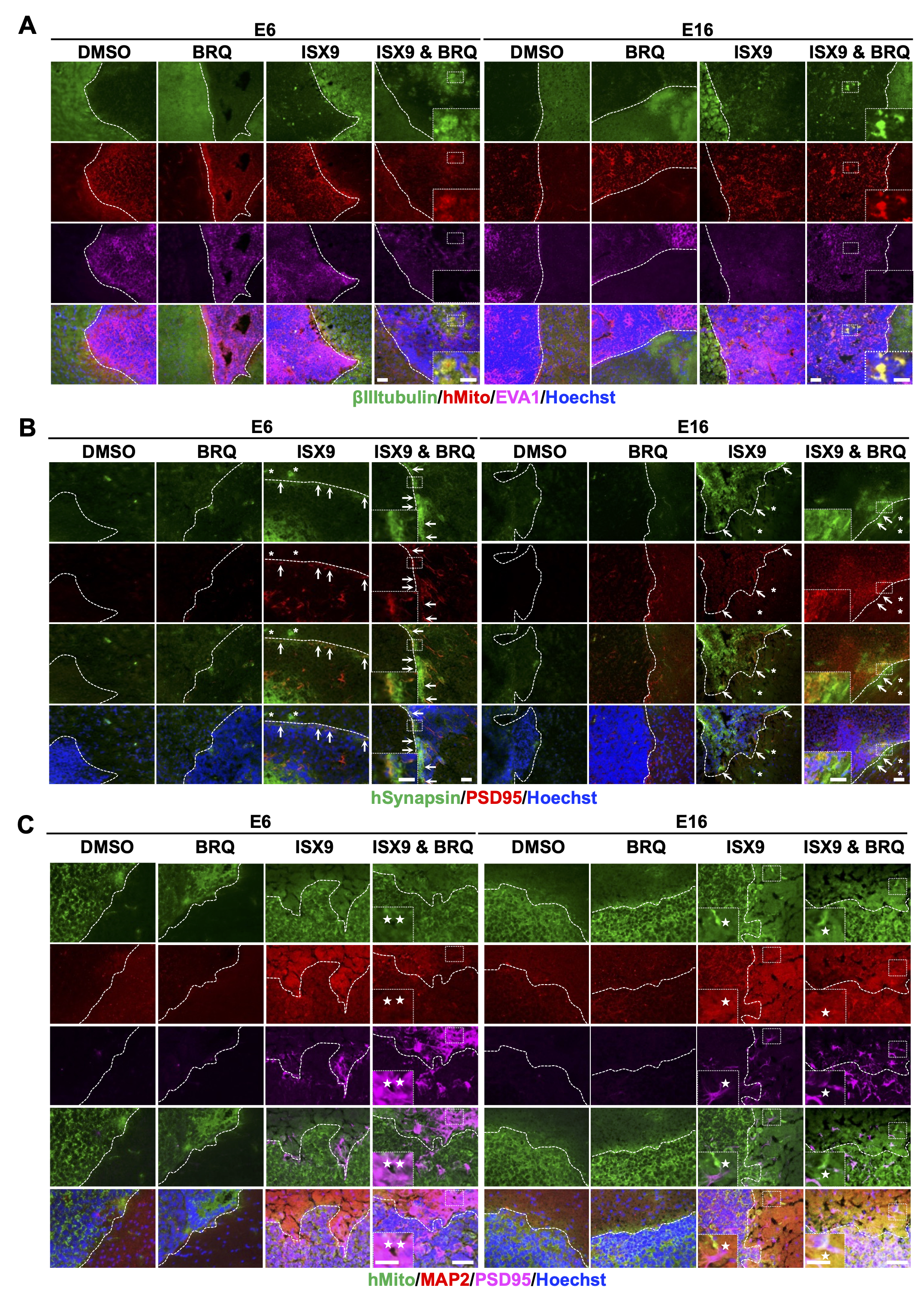

Supplement: Supplementary file 1 [file cells-15-00539-s001.zip › cells-4180682-Supplementary Figure S6.tiff]

## Supplemental Video

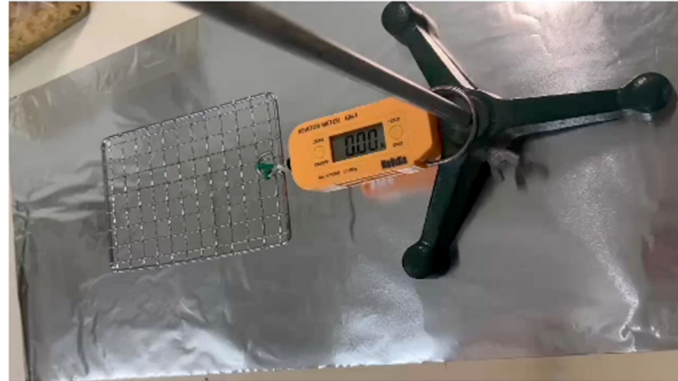

Supplement: Supplementary file 1 [file cells-15-00539-s001.zip › cells-4180682-Supplementary video.pdf]
